# Quality-of-life outcomes with endoscopic and microscopic type I tympanoplasty—a prospective cohort study

**DOI:** 10.1007/s00405-023-07938-6

**Published:** 2023-03-31

**Authors:** István Pap, Márton Kovács, Barbara Bölcsföldi, Zsolt Szakács, Imre Gerlinger, Bence Imreh, Alexandra Csongor, Vilmos Warta, István Szanyi

**Affiliations:** 1 Department of Otorhinolaryngology, Head and Neck Surgery (ENT), Medical School, University of Pécs, Munkácsy M. Street, No. 2, 7621 Pécs, Hungary; 2 Department of Languages for Biomedical Purposes and Communication, Medical School, University of Pécs, Pécs, Hungary; 3 First Department of Medicine, Medical School, University of Pécs, Pécs, Hungary; 4 Kanizsai Dorottya County Hospital, Nagykanizsa, Hungary

**Keywords:** Cosmetics, Endoscopy, Myringoplasty, Quality of life, Treatment outcome, Tympanoplasty

## Abstract

**Purpose:**

Endoscopic type I tympanoplasty was originally introduced in the 1990s and the extensive spread of this practice can be easily observed. The conventional technique performed involves the repair of a tympanic membrane perforation and is defined as microscopic type I tympanoplasty. The aim of this study is the comparison of quality-of-life (QoL) outcomes with endoscopic to that with microscopic type I tympanoplasty.

**Methods:**

All patients, or in the case of children with the aid of a parent, were asked to complete a novel QoL questionnaire drafted by our study group. The analysis was performed with descriptive statistics—mean, SD and relative frequency—and with a mixed model (generalized least squares fit). A two-sided *p* value of < 0.05 was regarded as statistically significant.

**Results:**

A total of 83 patients completed the questionnaire, 38 in the endoscopic group and 45 in the microscopic group. Every question represented a different. A statistically significant result was found in favor of the endoscopic approach regarding average hospitalization rate (*p* = 0.003) and cosmetic outcomes (*p* = 0.015). No statistically significant difference was otherwise observed between the groups.

**Conclusions:**

Based on our prospective cohort study, the QoL outcomes of endoscopic type I tympanoplasty in terms of postoperative pain, headache, nausea, vomiting, dizziness, taste disorder and hearing were comparable to the microscopic type I tympanoplasty. In regard to cosmetics, an increase in desirable results was achieved in the endoscopic group, particularly the average hospitalization rate proved to be statistically significantly lower than in the microscopic group.

## Introduction

The increased proportion among subjects suffering from chronic tympanic membrane perforations and, interestingly, those whom wish not to be operated on, suggests there is a considerable need for novel therapeutic procedures [1]. An additional systematic review found the incidence rate of chronic suppurative otitis media was 4.76‰, or nearly 31 million cases. Otitis media related hearing impairment has a prevalence of 30.82 per ten thousand. Annually, 21,000 individuals succumb due to complications of otitis media [2].

The microscopic tympanoplasty has been the standard procedure regarding the effective reconstruction of a perforated tympanic membrane, dating back to the middle of the twentieth century. This approach has some disadvantages caused by a retroauricular incision and the necessity in performing canaloplasty, primarily in cases of anterior perforation [3]. Endoscopic approach, however, is particularly advantageous in cases of anteriorly located, hard-to-see tympanic membrane perforations. In these cases, when microscopic approach was chosen at our institution, as well as many other otology surgeons often opted for a retroauricular incision. Since endoscopic ear surgery was first performed in the 1990s [4], it represents the ever-increasing, minimally invasive branch of otologic surgery. The endoscope is an ideal tool, enabling the surgeon to perform transcanal endoscopic ear surgery in its entirety, while the classical microscopic approach most likely requires an additional external incision, or a mastoidectomy [3]. In a study examining the prevalence of chronic mesotympanic otitis media among the adult population, it was found nine out of 1000 patients suffered from a perforated eardrum. Eight out of nine subjects disliked tympanoplasty for a variety of reasons [1] (e.g., cosmetic results, hospitalization).

When using an endoscope for visualization the need of an external incision, soft tissue dissection and bone removal can effectively be avoided. In addition, the consumption of medical resources can also be diminished resulting in a shorter rate of hospitalization [5]. This approach also provides a wider perspective in reference to enabling access into “hidden spaces” deep within the middle ear cavity.

The new technique was presented in Hungary for the first time at the Department of Otolaryngology and Head and Neck Surgery of the University of Pécs, which was preceded by a series of international courses including cadaver dissections.

In reference to further scientific support of the above-mentioned declarations, our study group conducted a meta-analysis comparing the two approaches, analyzing different surgical outcomes in regard to type I tympanoplasty [5].

In analyzing our data, we noted the majority of included studies did not take into account factors which influence the patient’s quality of life. The notion of quality of life (QoL) first appeared in the 1970s as a new important outcome to be analyzed in healthcare [6]. The World Health Organization (WHO) defines Quality of Life as an individual's perception of their position in life in the context of the culture and value systems in which they live and in relation to their goals, expectations, standards, and concerns [6].

The measurement of QoL in published literature is usually performed using preexisting questionnaires adapted to different patient groups and languages depending on the illness and population being analyzed.

The majority of existing QoL questionnaires examining patient population with an existing ear pathology focuses on the outcomes regardless of the used surgical technique. In addition, these studies mostly compare a healthy group of patients to a group of patients afflicted with chronic otitis media.

Post-operative quality of life aspects are relevant outcomes to evaluate when also comparing two surgical techniques, in particular, if they compare a new minimal-invasive technique with the traditional more invasive technique. Our study group concluded a questionnaire which compares two surgical approaches, respectively endoscopic type I tympanoplasty with the microscopic type I tympanoplasty, regarding quality-of-life outcomes warrants development.

This led us to the determination of drafting such a quality-of-life questionnaire and performing a prospective study.

## Materials and methods

### Ethical considerations

Prior to conducting the study, ethical approval was accorded from the *Blinded for Review* and fully adheres to the Declaration of Helsinki Ethical Principles for Medical Research Involving Human Subjects. Ethical approval no. 8107 PTE-2019.

### Questionnaire development and structure

The formulation of the questionnaire was performed by our work team based on other otologic surveys and our own clinical experience [6–8]. Since the study was conducted in Hungary, the primary language was Hungarian, after which, due to the process of validation and international publication, it was later translated into English.

The translation was validated by the Department of Languages for Biomedical Purposes and Communication, *Blinded for Review*, and assigned validation code number: 04/11/2021/1 (Table 1).

**Table 1:** Questions included in the questionnaire

1. Have you experienced pain at the site of surgery following the operation? If so, please rate on a scale from 1 to 10. 1 is the mildest pain you experience, and 10 is the most intense pain you experienced
2. Did you experience a headache following surgery, presumably as a result of the operation? If so, please rate it on a scale from 1 to 10, according to the rating above
3. Have you experienced nausea as a result following surgery?
4. Did vomiting occur following surgery?
5. Did you experience dizziness following surgery? *If you receive the questionnaire at least 4 months following the operation, the question will change to the following:* Is dizziness more common since the operation?* Please explain the quality of dizziness, including its nature, frequency, and duration.*
6. Have you experienced a taste disorder following surgery? *If you receive the questionnaire at least 4 months after the operation, the question will change to the following:* Do you experience a taste disorder due to the operation? *Please explain the quality of the change in taste, including its localization (where?), The sense of taste (which taste?), The direction of change (strengthened/weakened?)*
7. Are you satisfied with the cosmetic results following wound healing? Please rate on a scale from 1 to 10. A value of 1 expresses complete dissatisfaction with wound healing, while a value of 10 suggests a nearly invisible scar
8. How many days were you hospitalized following surgery?
9. How many days of recovery following surgery prior to your return to work?
10. Did you observe a change in your hearing experience following surgery*? If you receive the questionnaire at least 4 months following the operation, the question is regarding your current status*

### Patient selection

All patients enrolled in the study were operated on at the Department of Otolaryngology, Head and Neck Surgery, *Blinded for Review*. Only patients who underwent type I tympanoplasty were considered eligible for inclusion.

To rule out as much as possible the heterogeneity of the database regarding surgeon’s experience, we enrolled only patients who were operated by surgeons with at least 10 years of experience performing ear surgery.

All surgeries were performed under general anaesthesia.

The onset of the study was 4 April 2017. The last patient enrolled underwent surgery in November 2021. Demographic data were also collected. Inclusion and exclusion criteria for the study are listed below and are available for both the endoscopic and the microscopic group.

*Inclusion criteria*

1. Patients who were afflicted with chronic mesotympanic otitis media
2. The affected side to be operated on must be dry (inactive) at least 1 month prior to surgery

*Exclusion criteria*

1. Patients with extended attical disease or cholesteatoma
2. Patients with active discharging ear
3. Patients with severe chronic conditions (such as diabetes mellitus)
4. Patients requiring revision surgery
5. Patients who underwent previous ear surgery

### Data collection

Main workflow stations are depicted in the below inserted flowchart.
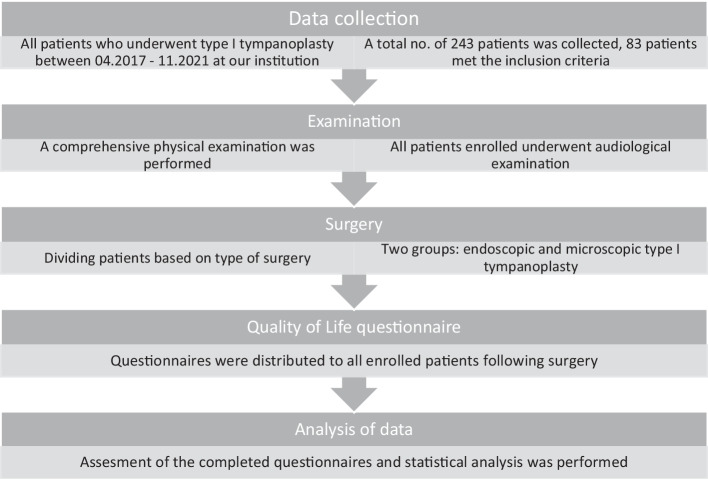


We concluded a prospective cohort study. In all cases, the consent form was signed prior to surgery. All patients, or in the case of children with the aid of a parent, were asked to complete the QoL questionnaire drafted by our study group. Questionnaires were distributed in the first 24 h following surgery to accurately depict the patient’s postoperative quality of life.

Patients were asked to complete the questionnaire within 1 week following surgery, or until their first follow-up visit following surgery.

The questionnaire was redistributed at least 4 months following the operation.

In the follow-up study, we repeatedly asked for feedback from patients regarding dizziness, taste disturbance and hearing loss. Dizziness and taste disturbance were again qualitatively included in the table and assigned a value of 0 or 1, depending on whether the patient experienced any of these present complaints. The change in hearing according to whether it modified in a negative direction (0), stagnated (1), or in a positive direction (2) was included in the table.

Audiological examinations were also performed not only prior to surgery, but also during the follow-up visit at 1 month and at least 4 months following surgery. The data used for statistical analysis were the result obtained at 1 month post-operatively.

Our study group also examined the recovery rate of the patients. The question arose as to how many days patients needed before they can return to work following surgery. In the case of students, it was a question of returning to school, while in the case of retired patients, the question reflected to the days needed for returning to a normal daily rhythm.

The learning curve suitably represents the change in surgical times. In our case, the duration of surgery is expected to decrease as the time progresses. It is an accepted fact in which one of the setbacks regarding endoscopic ear surgery is the one-handed dissection. The surgeon holds the endoscope on one hand and can only use the other hand for preparation. In contrast, both hands can be used when using a microscope.

## Analysis of data

The analysis was performed with descriptive statistics—mean, SD and relative frequency—and with a mixed model (generalized least squares fit). A two-sided *p* value of < 0.05 was regarded as statistically significant. Statistical analyses were performed using RStudio Team (2020, RStudio: Integrated Development for R. RStudio, PBC, Boston, MA URL http://www.rstudio.com/).

## Results

### Characteristics of the patients included

A total of 83 patients completed the questionnaire, 38 in the endoscopic group and 45 in the microscopic group (Table 2).

**Table 2:** Representation of basic population characteristics

	Endoscopic group	Microscopic group
Total no. of patients (female/male)	38 (17/21)	45 (24/21)
Average age (mean, range in years)	43 (12–70)	51 (8–74)
Right sided surgeries (no.)	15	34
Left sided surgeries (no.)	23	11

### Outcomes

Regarding post-operative pain, we found a slight, statistically non-significant difference in favor of the endoscopic group.

When examining the results of post-operative headache, we found no representative differences.

**Fig. 1:**
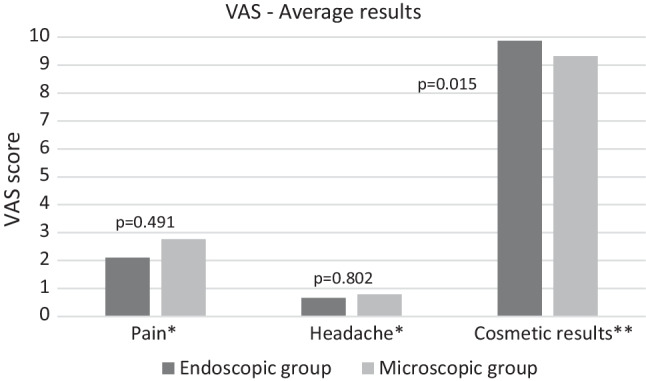
Results of the first three questions based on a VAS. *Patients were able to rate their specific symptom, for example, pain experienced in the postoperative hours on a visual analog scale of up to 10. A value of 10 indicated the most intense pain the patient experienced. **A visual analog scale of up to 10 was used to assess how satisfied the patient was with the cosmetic appearance of the surgical scar. A value of 10 represented maximum satisfaction

Since endoscopic type I tympanoplasty is proven to be a less invasive procedure, our main focus was on examining cosmetic results. Here, a statistically significant difference was found in favor of the endoscopic group (Fig. 1).

Audiological examinations where also performed not only prior to surgery, but also during the follow-up visit at 1 month and at least 4 months following surgery. The data presented in this manuscript are the results obtained at 1 month post-operatively.

**Fig. 2:**
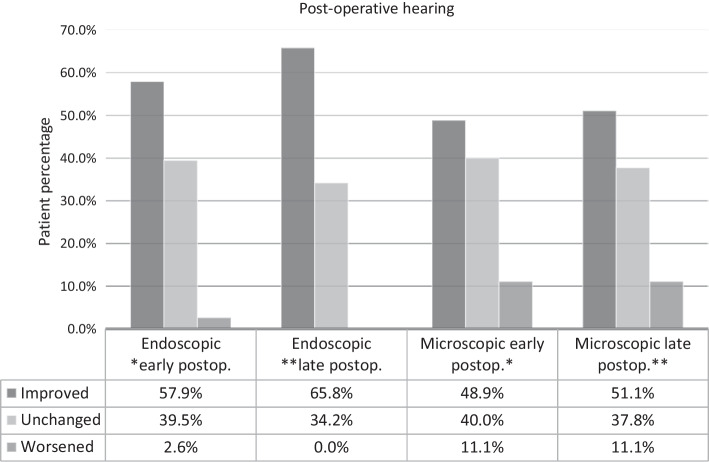
Change in post-operative hearing results. *Early postoperative infers completion of the questionnaire was performed in the first 24 h following surgery. **Late postoperative infers the data used for statistical analysis was collected 1 month following surgery

Regarding this question, we were curious if the patient had experienced a change in their hearing following surgery, and if so, in what direction. In analyzing our data, we found no statistically significant difference between the two groups. However, a minimal benefit in favor of endoscopic technique was observed (Fig. 2).

In analyzing the incidence of post-operative dizziness (*p* = 0.962) and taste disorder (*p* = 0.769), we found no compelling difference between the two approaches. It is worth mentioning, some patients reported a change in taste in the “Note:” option, according to which the perception of sweet taste had significantly increased at the expense of other tastes.

In examining the incidence regarding post-operative nausea, no statistically significant difference was observed (*p* = 0.135), although we obtained better mean values in the endoscopic group.

We did not find a statistically significant difference between the values of the two groups when examining the presence of post-operative vomiting (*p* = 0.790).

**Fig. 3:**
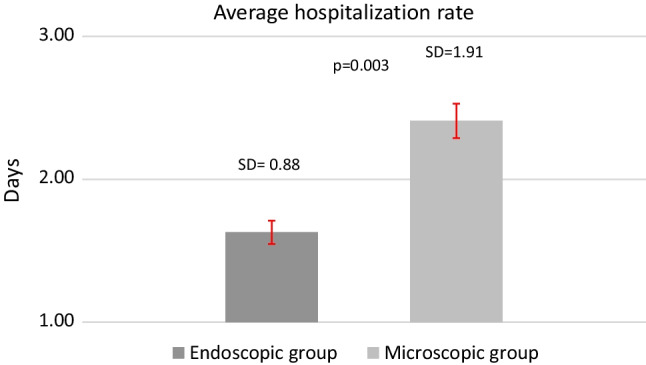
Representing the difference between the two groups, regarding average hospitalization rates

Patients were asked to declare how many days they were hospitalized following surgery. A statistically significant difference was found between the values of the two groups in favor of the endoscopic group (Fig. 3).

### Evaluation of the questionnaire completed during follow-up

In this subsection, the values of the three variables: dizziness, taste disturbance and post-operative hearing of the endoscopic group are compared. Late post-operative hearing results are depicted in Fig. 2.

**Fig. 4:**
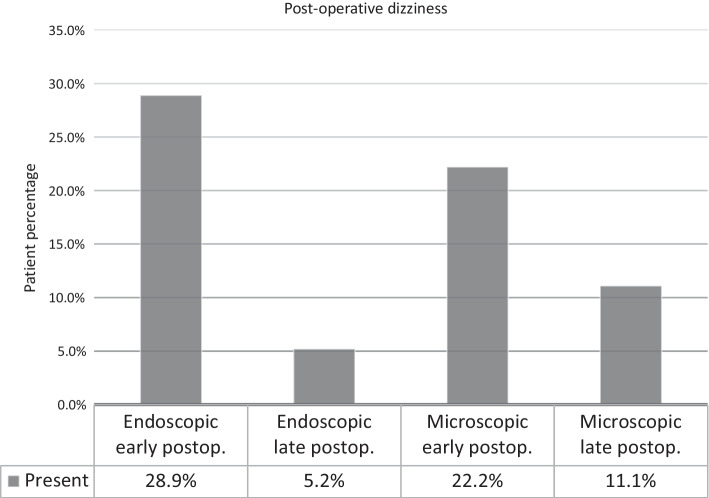
Depicting the incidence rate of post-operative dizziness

In the control study, only two patients continued to complain of dizziness. As can be seen, the proportion of those who complained of dizziness decreased significantly. This implies a statistically significant difference. The *p* value is calculated for late post-operative data (*p* = 0.025). Five patients who complained of dizziness following surgery were eventually free of complaints (Fig. 4).

**Fig. 5:**
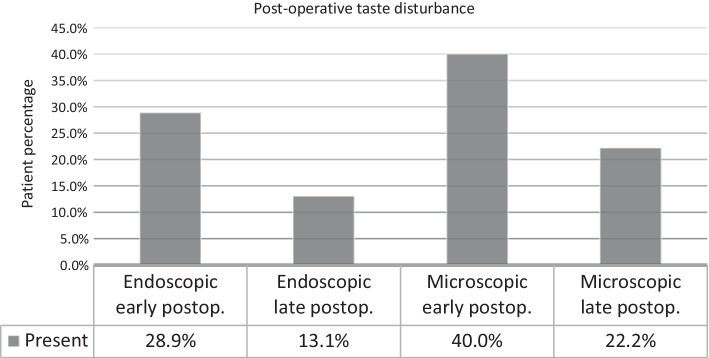
Representing the incidence of post-operative taste disturbance

Only four patients continued to complain of taste disturbances. We found the perception in taste improved, at a significance value of *p* = 0.046, a statistically significant difference was confirmed between the early and late post-operative results. Four patients who had previously complained of taste disturbances eventually reported free of complaints (Fig. 5).

## Discussion

Chronic otitis media is a disease which can be associated with a high morbidity and affects approximately 2% of the population, of which, when tallied, highlights a seriously high number of patients worldwide [7].

There has been a growing interest in developing questionnaires or other instruments which aids medical research towards defining surgical and non-surgical outcomes from a patient’s perspective. These outcome measurements play an important role in research. Questionnaires are considered to be a valid and effective tool for data collection.

Admittedly, only a relatively small number of QoL questionnaires exist in the literature which are used for analyzing a patient’s life quality suffering from chronic otitis media (COM).

The COMQ-12 has been developed with the objective of evaluating overall burden of disease from the perspective of the patient [7]. This questionnaire compares the scores of normal, healthy patients to those patients afflicted with COM.

Phillips et al. found to varying degrees, the validity of a study is dependent upon how one defines the “normal” population [7].

Berat et al. adapted the COMQ-12 into the Turkish language and conducted a study comparing the data collected from 100 healthy and 100 patients suffering from COM [8]. They unveiled a statistically outweighed difference between the cholesteatoma and without cholesteatoma groups in regarding total scores. In addition, they concluded the COMQ-12 can be a useful tool to differentiate chronic otitis media, with or without cholesteatoma.

Another predominantly used questionnaire is the Chronic Ear Survey (CES). The CES is an instrument to measure the impact of chronic otitis media and its treatment. The survey provides information regarding total ear-specific health, including subscore information regarding activity restriction, symptoms and medical resource usage attributable to chronic otitis media [9]. Nadol et al. found the 147 patients enrolled in the prospective, non-randomized study suffering from chronic otitis media have significantly decreased CES scores compared with the unaffected control group and surgical intervention provides a major improvement in ear-specific outcomes [10]. Ralli et al. also adapted the CES into Italian [6].

Devi et al. conducted a study evaluating the effect of type I tympanoplasty on the QoL of patients suffering from COM (safe type) [11]. They used a Modified Chronic Otitis Media-4 questionnaire [12]. The analyzed parameters included the following, physical suffering, hearing loss, emotional distress, and activity limitation. In conclusion, they found type 1 tympanoplasty considerably improves the quality of life among patients in terms of physical suffering, hearing loss and emotional distress, postoperatively. There was representative correlation between preoperative and postoperative scores. Their research also concluded there was statistically significant improvement in hearing, postoperatively.

In concluding from the above citated articles, using questionnaires in different languages ushers an opportunity to study and compare different populations and cultures, acquire information from various health systems to understand the importance of a disease and its treatment adequacy.

Thus far, the questionnaires we analyzed focused either on comparing a healthy population sample with a number of patients suffering from COM, or they concentrate on examining the burden of the disease which it exerts upon the patient’s quality of life.

Notably, none of the above-mentioned articles and questionnaires investigated the impact of the surgical technique regarding the patient’s quality of life. The questionnaire conceived by our workgroup focuses on analyzing how the chosen surgical technique influences the patient’s post-operative quality of life. In our perspective, it can also be implemented in a patient population with any kind of COM who underwent surgery.

In this study, we focused on patients who experienced type I tympanoplasty regarding chronic mesotympanic otitis media. Our goal was to emphasize the role of endoscopic ear surgery.

We found that in reference to post-operative pain, headache, nausea and vomiting, there was no statistically significant difference between the two groups, despite a slight advantage in favor of the endoscopic technique which we observed. All enrolled patients underwent general anaesthesia with the same medication protocol used at our institution. Regarding post-operative nausea it is worth mentioning the result of this outcome may be affected by individual drug reaction.

With regards to post-operative dizziness and taste disorder, the questionnaire was completed by patients in the immediate post-operative period, but also at follow-up and at least 4 months following surgical intervention. We found no statistically significant difference between the two groups; however, in the late post-operative period, the results were in favor of the endoscopic group.

During the evaluation of cosmetic results, we used a visual analog scaling method as described in the material and method chapter. Our analysis expressed a statistically significant outcome in favor of the endoscopic type I tympanoplasty. This result concurs with our previous findings considering this outcome in our meta-analysis [5].

In examining the hospitalization rate, we also found the number of days spent hospitalized following this type of surgery is statistically significantly lower in the endoscopic group when compared with the patient population who underwent microscopic tympanoplasty. In our opinion, this is an important outcome, since there is an increasing emphasis on minimally invasive surgery and how it correlates with the economic analysis of health care.

In reference to the recovery rate of patients, the question arose as to how many days patients needed before they can return to work following surgery. However, we found these results are not so easily interpreted. There are underlying socio-economic factors which can influence the patient’s decision to return to their job. For example, it is not conventional for a patient to be unable to return to work for 90 days due to undergoing type I tympanoplasty, without any postoperative complications. Identifying this we decided not to include the statistical analysis of the above outcome in this paper.

In addition, post-operative hearing was also examined. We performed the statistical analysis also based on pure tone audiometry thresholds; however, since this study was aiming at QoL aspects, we decided to include only the results given by the patients in the questionnaire. It was an option of deciding whether the patients hearing remained the same, worsened or became better. We did not find any statistically significant difference between the two groups, although in the immediate and late post-operative period a slight percentage advantage in favor of the endoscopic group was observed.

The learning curve suitably represents the change in surgical times. In our case, the duration regarding surgery is expected to decrease as the time progresses. This tendency is hampered by a factor in which one of the setbacks to using the endoscope is one-handed dissection.

This study has a limitation in which this trial was performed at a single institution, and further studies are encouraged to be conducted in more institutions. Due to this aspect, our long-term plans include international and national cooperation with other institutions in the examination of the questionnaire we compiled.

Endoscopic ear surgery represents the ever-increasing, minimally invasive branch of otologic surgery. New results culminating in the recent past comparing the two approaches surgical outcomes warranted a study measuring the novel methods impact on the life quality of the patients.

## Conclusion

Our findings indicate the endoscopic approach competitively achieved equally as well results regarding the evaluated quality of life outcomes as the microscopic method. In fact, regarding cosmetic outcomes and average hospitalization rate, in referencing our study, the endoscopic approach led to statistically significant better results.

Hence, the implementation of endoscopic type I tympanoplasty regarding the treatment guidelines of chronic mesotympanic otitis media is strongly worth considering.

## Data Availability

The data that support the findings of this study are available on request from the corresponding author. The data are not publicly available due to privacy or ethical restrictions.
